# High-Throughput Analysis Reveals Seasonal Variation of the Gut Microbiota Composition Within Forest Musk Deer (*Moschus berezovskii*)

**DOI:** 10.3389/fmicb.2018.01674

**Published:** 2018-07-26

**Authors:** Xiaolong Hu, Gang Liu, Yimeng Li, Yuting Wei, Shaobi Lin, Shuqiang Liu, Yunlin Zheng, Defu Hu

**Affiliations:** ^1^College of Animal Science and Technology, Jiangxi Agricultural University, Nanchang, China; ^2^Laboratory of Non-invasive Research Technology for Endangered Species, College of Nature Conservation, Beijing Forestry University, Beijing, China; ^3^Institute of Wetland Research, Chinese Academy of Forestry, Beijing, China; ^4^Zhangzhou Pien Tze Huang Pharmaceutical, Co., Ltd., Zhangzhou, China

**Keywords:** bacterial ecology, symbioses, diet, seasonal variation, *Moschus berezovskii*

## Abstract

The gut microbiota plays a key role in the nutritional ecology of ruminants, and host diet has a significant effect on these microbial communities. Longitudinal studies assessing variation of seasonal microbiota in animals can provide a comparative context for interpreting the adaptive significance of such changes. However, few studies have investigated the effects of seasonally-related dietary shifts on the gut microbial communities of endangered forest musk deer (FMD), and the national breeding programs need this information to promote the growth of captive populations. The present study applied bacterial 16S rRNA genes based on high-throughput sequencing to profile the fecal microbial communities of FMD across four seasons. Microbial diversity was higher in seasons with dry leaf diets (winter and spring) compared to seasons with fresh leaf diets (summer and autumn). The dominant microbial phyla were Firmicutes and Bacteroidetes, and the core bacterial taxa also comprised mostly (94.40% of shared OTUs) Firmicutes (37 taxa) and Bacteroidetes (6 taxa), which were relatively stable across different seasons. The Firmicutes–Bacteroidetes ratio declined in seasons with fresh leaf diets relative to seasons with dry leaf diets, and the dominant genera among the four seasons showed no significant variation in abundance. This work explores the seasonal variation in the microbial communities of FMD for the first time, and reveals how gut microbial community dynamics vary seasonally in accordance with differences in dietary plants (fresh and dry leaf). These results indicate that the annual cyclic reconfiguration of FMD gut microbiota could be associated with shifts in dietary nutrients, which is important information to inform captive FMD management.

## Introduction

The digestive tracts of animals are complex microecosystems that include gut microbiota that exist in a dynamic symbiosis between host, microbiota, and the environment (Lee et al., 2013). Gut microbiota plays a pivotal role in the growth (Schwarzer et al., 2016), development (Sommer and Bäckhed, 2013), and immunity (Fung et al., 2017) of the host. Further, opportunistic pathogens within the gut microbiota community can invade hosts through the gastrointestinal tract (Roeselers et al., 2011). Host–microbiota relationships have garnered increased research attention in recent decades, particularly because the advent of high-throughput sequencing technologies have allowed more efficient and rigorous investigations of gut microbiota. Indeed, these methods have been employed by lots of studies to explore the microbiota of human beings (Zhang et al., 2014; Smits et al., 2017), carnivores (Xue et al., 2015; Wu et al., 2017), primates (Amato et al., 2013; Sun et al., 2016), rodents (Stevenson et al., 2014; Maurice et al., 2015), birds (Hird et al., 2015; Kreisinger et al., 2017), and ruminants (Gruninger et al., 2014; Cersosimo et al., 2015). Ruminants rely on mutualistic gut microbiota to harness energy via the fermentation of indigestible dietary material that includes cellulose and hemicellulose (Thoetkiattikul et al., 2013), and thus gut microbiomes contribute significantly to the nutrition and health of ruminants.

The colonization of the gut by the microbiome can be affected by many factors, but diet is one of the important factors in shaping the microbial composition and function of microbiota (Ley et al., 2008; Muegge et al., 2011). Different nutrients within diets (e.g., fiber, fats, or starch) select for different microbial taxa, and dietary changes can rapidly affect the composition of gut microbiota, although communities can also be remarkably stable (Faith et al., 2013; Amato et al., 2015; Glenwright et al., 2017). For most wild mammals, dietary shifts across seasons are common (Amato et al., 2013; Ren et al., 2016). Seasonal changes often result in changes of food availability and dietary compositions, thereby shifting the energy intake of hosts, and subsequently, the gut microbiota (Bergmann et al., 2015; Trosvik et al., 2018). For example, high fiber diets can result in increased abundances of taxa capable of degrading fiber, in addition to efficient fermenters of insoluble carbohydrates, as noted during periods of low food quality for giant panda (Williams et al., 2012; Wu et al., 2017). These diet-related changes may increase energy extraction from foods and consequently alter host metabolic pathways. Thus, alteration of gut microbiota function may be a mechanism by which animals compensate for reduced energy intake (Sommer and Bäckhed, 2013; Sommer et al., 2016; Springer et al., 2017). Conversely, increased disease and reduced survival may occur resulted from resource limitation, on condition that the gut microbiota fails to respond to these dietary alterations or impacts digestive efficiency negatively (Gogarten et al., 2012).

Forest musk deer (*Moschus berezovskii*; FMD) are small ruminants widely distributed in mountains and forests of East Asia, with China being the key parts of its range (Meng et al., 2006). The adult male FMD can secrete musk, which is a remunerative raw material used in the traditional Chinese medicine and perfume industry. In order to provide a sustainable musk supply and protect endangered FMD populations, which has suffered a steep decline due to habitat destruction and over-exploitation, China government launched the breeding programs in the 1950s (Meng et al., 2006). Nevertheless, the population size of captive FMD has remained small to date, and gastrointestinal diseases are an important constraint to population growth (Yan et al., 2016). However, the relationship between gastrointestinal disease and FMD gut microbiota remains unclear. Captive FMD are fed supplementary concentrates, whereas the primary food of musk deer are natural leaves. Nevertheless, the energy compensation capacity of *Moschus* gut microbiota has not been tested in the context of seasonal variation in diet. Moreover, little is known about the gut microbiota of captive wild ruminants. In this study, we characterized the seasonal variation of gastrointestinal microbiota in captive FMD using high-throughput sequencing of 16S rRNA genes from fecal samples. Further, we tested the hypothesis that FMD gut microbiota could compensate for low food quality due to seasonal variations in diet. Thus, this investigation represents a comprehensive analysis of the dietary-related seasonal variation of FMD gut microbiota structure, and it contributes significantly to studies of gut microbial dynamics within wild and captive ruminant species.

## Materials and Methods

### Sampling Sites and Animal Diets

The breeding center of FMD used for sampling in this study is in Baoji, Shaanxi Province, China (34°28′ N, 106°78′ E). The area belongs to a region of the southern Qinling Mountains at an altitude of 1,500 m, with an annual average temperature of 11.3°C and average annual rainfall of 634.6 mm. The Qinling Mountains are ideal for establishing FMD breeding centers because of their abundant biodiversity, rich natural food sources, and suitable climate. Leaves collected from the natural habitat of FMD were the main food, and animals were fed fresh leaves in summer and autumn (April–September), and dried leaves in winter and spring (October–March). The leaves were sourced from the following species: *Ulmus pumila*, *Usnea diffracta*, *Picrasma chinensis*, *Swida bretschneideri*, *Anacardiaceae rhus*, *Fraxinus chinensis*, *Morus alba*, *Acer mono, Schisandra chinensis*, and *Clematis armandii*. Several types of concentrated feed (soybean flour, wheat bran, corn flour) were supplemented to keep the carbohydrate levels suitable for normal fermentation in the rumen, and seasonal vegetables and fruits were used as food supplements to provide vitamins. Water was provided *ad libitum*.

### Samples Collection

Forest musk deer were isolated all night to be able to collect samples from each individual. A total of 32 adult FMD individuals were sampled in spring (collected in March, SP1–SP8), summer (collected in July, S1–S8), autumn (collected in September, A1–A8), and winter (collected in December, W1–W8). All experimental animals are healthy and ear labels were used to differentiate individuals. Feces within housing was removed every evening (18:00–20:00), so that fresh samples could be collected the next morning. Fresh fecal samples were immediately stored in liquid nitrogen and transported to the laboratory using a mobile refrigerator, then frozen at -80°C until DNA extraction within 12 weeks. This study was carried out in accordance with the recommendations of the Institution of Animal Care and the Ethics Committee of Jiangxi Agricultural University and Beijing Forestry University. The protocol was approved by the Ethics Committee of Jiangxi Agricultural University and Beijing Forestry University.

### DNA Extraction

The extraction of bacterial DNA in feces was performed using QIAamp DNA Stool Mini Kit (QIAGEN, Hilden, Germany) according to the official protocol with minor modifications in fecal pretreatment (crush samples in dry ice). The quality of fecal DNA was tested visually by 1.0% agarose gel electrophoresis. The concentration and purity of DNA were tested using Qubit dsDNA HS Assay Kit (Life Technologies, Carlsbad, CA, United States). Finally, the extracted DNA was preserved at -80°C until PCR.

### PCR Amplification and High-Throughput Sequencing of 16S rRNA Genes

Purified DNA was used as template for PCR amplification of 16S rRNA gene fragments. The hypervariable V3/V4 regions of 16S rRNA genes were amplified using universal bacterial PCR primers 341F and 805R (Jakobsson et al., 2014). The Miseq adapters (**Supplementary Table S1**) were added to the ends of primers to qualify primers for downstream next generation sequencing (NGS) sequencing. The PCR reaction mixture and two-step PCR procedures were in line with our previous study (Hu et al., 2017). After the PCR products were purified, high-throughput sequencing was performed using the MiSeq PE300 chemistry on the Illumina MiSeq platform (Illumina, San Diego, CA, United States) at Sangon Biotech in Shanghai.

### Statistical and Bioinformatics Analysis

The software QIIME 2 was used to control sequence quality. The sequencing reads that did not meet the following criteria are discarded: length >200 bp, mean quality score ≥20, homopolymers ≤8 bp, no ambiguous bases. Then the PCR-based errors and chimeric sequences were removed from database using UCHIME algorithm. Operational taxonomic units (OTUs) were defined using the VSEARCH 1.9.6 (Rognes et al., 2016) referring to the Silva 119 database at 93–97% sequence identity. Distance matrices were then constructed with OTU representatives using the dist.seqs command of Mothur (Schloss et al., 2009). The sequencing depth (Good’s coverage) and alpha diversity indexes (ACE, Chao1, Shannon, and Simpson) were calculated using Mothur (Schloss et al., 2009).

The Ribosomal Database Project (RDP) classifier (Cole et al., 2005) against Silva 119 database was applied to taxonomic classification of OTUs at confidence threshold of 0.8. Non-parametric Kolmogorov–Smirnov (K–S) tests were used to test for data normality. One-way ANOVA (for normally distributed data) and non-parametric Kruskal–Wallis ANOVA (for skewed distributed data) were used to compare the relative abundances of the five most abundant phyla and genera among four seasons. All above tests were performed using the SPSS software package vs. 20.0 (IBM Corp., New York, NY, United States). Non-metric multi-dimensional scaling (NMDS) ordination based on Bray-Curtis dissimilarities of OTU composition among samples was used to visibly identify community differences, and an analysis of similarity (ANOSIM) test was used to assess the statistical significance of community compositional differences among groups by PRIMER 6.0 (Clarke and Gorley, 2006). Heatmap analysis based on the Bray–Curtis dissimilarity with average linkage hierarchical clustering was performed to test whether the samples within each group could be clustered together based on bacterial community composition using gplots package in R^1^. Venn diagrams and pie charts were used to visibly determine the OTUs shared by all group members that were then defined as the core microbiome using VennDiagram package in R. The LDA histogram and cladogram were generated using the LEfSe program^2^. The raw sequences obtained in this study were available through the NCBI Sequence Read Archive (accession number SRR5196686).

## Results

### Quality Filtering and Validation of the Dataset

After quality filtering, a total 378,511 reads of 16S rRNA gene (mean length = 408 bp) were retained and each sample totaled between 21,183 and 51,412 (mean = 34,343 ± 8,195; **Supplementary Table S1**). The Good’s coverage estimates of 32 samples were ranging from 73.95 to 89.60% (mean = 81.88%; **Supplementary Table S2**), suggesting that more than 80% of the diversity estimated in the samples were recovered. A total of 124,260 OTUs were obtained, with samples harboring an average of 8,386 ± 2,229 OTUs (range: 4,786–13,780; **Supplementary Table S2**). Rarefaction curves of Shannon index values indicated that the bacterial diversity of each sample was adequately measured at present sequencing depth, through rarefaction curves of observed OTUs showed a continuous rise with more sequencing (**Supplementary Figure S1**). The percentage of OTUs unassigned to genus level range from 11.87 to 38.77% in 32 samples, and accounted for 27.12% of the total OTUs. Taxonomic classification of OTUs resulted in a total of 39 phyla and one unclassified group across the dataset.

### Core Bacterial Communities in FMD Across Four Seasons

**FIGURE 1:**
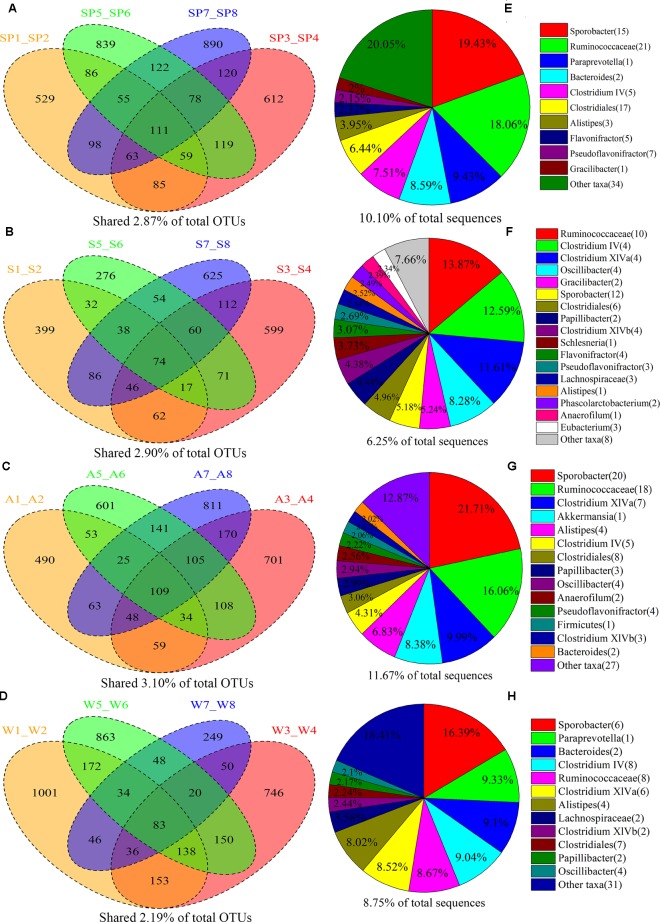
Sharing of OTUs and bacterial taxa within the four seasonal sample groups. The Venn diagrams shows the numbers of OTUs (97% sequence identity) that were shared or unshared by the individuals of forest musk deer sampled at spring **(A)**, summer **(B)**, autumn **(C)**, and winter **(D)**, respectively, depending of overlaps. For presentation two individuals had to be combined (e.g., SP1_SP2) thereby reflecting the number of OTUs shared by those two individuals. The pie diagrams show the core bacterial composition of groups corresponding to the left Venn diagrams: **(E)** spring, **(F)** summer, **(G)** autumn, and **(H)** winter. The taxa that occurred at abundance lower than 2% were included as “Other taxa.”

The shared taxa among all individuals within sample groups were considered as core bacterial communities. The number of OTUs shared by all individuals within each group was 111, 74, 109, and 83 in the spring, summer, autumn, and winter, respectively (**Figure 1**). The core bacterial families in the spring were *Ruminococcaceae*, *Prevotellaceae*, *Bacteroidaceae*, *Lachnospiraceae*, *Rikenellaceae*, *Gracilibacteraceae* (**Supplementary Figure S2B**); and *Ruminococcaceae*, *Lachnospiraceae*, *Gracilibacteraceae*, *Rikenellaceae*, *Acidaminococcaceae*, *Eubacteriaceae* for the summer (**Supplementary Figure S3B**); and *Ruminococcaceae*, *Lachnospiraceae*, *Verrucomicrobiaceae*, *Rikenellaceae*, *Bacteroidaceae* for the autumn (**Supplementary Figure S4B**); and *Ruminococcaceae*, *Lachnospiraceae*, *Prevotellaceae*, *Bacteroidaceae*, *Rikenellaceae* for the winter (**Supplementary Figure S5B**). Most of the core families (94.40% of shared OTUs) belonged to Firmicutes (37 taxa) and Bacteroidetes (six taxa). At the class level, the core gut bacterial taxa of FMD mainly included *Clostridia* and *Bacteroidia* (**Supplementary Figures S2D**–**S5D**) in addition to *Clostridiales* and *Bacteroidales* (**Supplementary Figures S2C–S5C**) at the order level. At the genus level, core bacteria mainly included the *Sporobacter*, *Paraprevotella*, *Bacteroides*, *Clostridium IV*, *Clostridium XlVa*, *Alistipes*, *Oscillibacter*, *Gracilibacter*, and *Akkermansia* (**Supplementary Figures S2A–S5A**).

### Seasonal Variation of Gut Bacterial Communities in FMD

**FIGURE 2:**
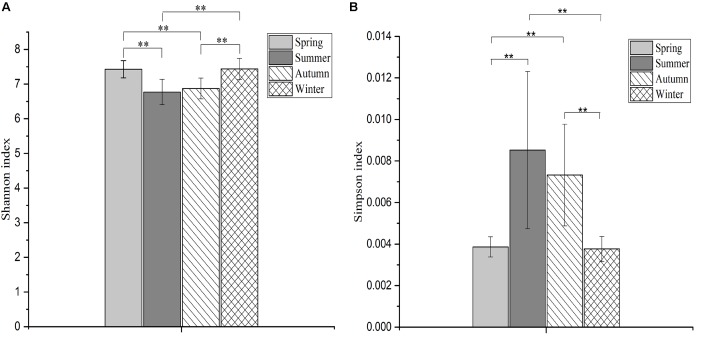
The differences in microbial diversity (Shannon and Simpson) among four seasons. The histogram **(A)** showing the differences in Shannon index among four seasons, and the plot **(B)** showing the differences in Simpson index among four seasons. The significances of Shannon and Simpson indices were determined using the One-way ANOVA and non-parametric Kruskal–Wallis tests, respectively. ^∗∗^Means that an extremely significant difference was found.

The Shannon index of communities in spring and winter were significantly higher than in summer (spring, *F* = 10.68, *df* = 3, *p* < 0.001; winter, *F* = 10.68, *df* = 3, *p* < 0.001) and autumn (spring, *F* = 10.68, *df* = 3, *p* = 0.001; winter, *F* = 10.68, *df* = 3, *p* = 0.001; **Figure 2A**). The Simpson index of communities in spring and winter were significantly lower than in summer (spring, H = 23.36, *df* = 3, *p* = 0.004; winter, H = 23.36, *df* = 3, *p* = 0.008) and autumn (spring, H = 23.36, *df* = 3, *p* = 0.002; winter, H = 23.36, *df* = 3, *p* = 0.004; **Figure 2B**).

**FIGURE 3:**
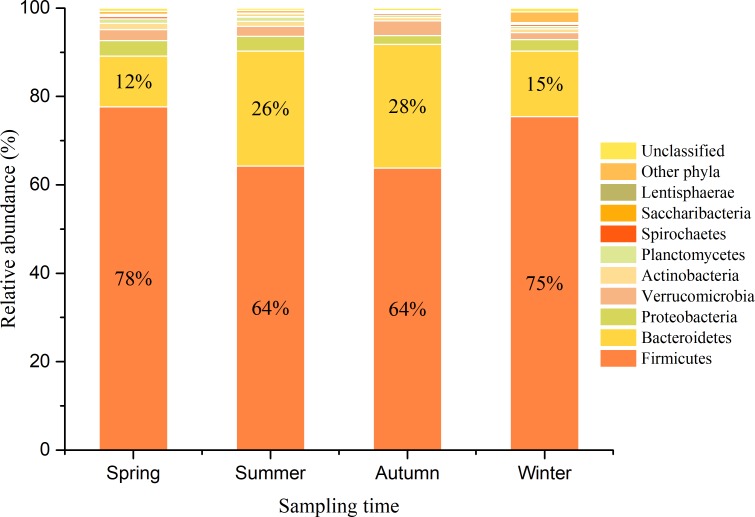
Bacterial composition of FMD in four seasons at phylum level. The histograms represent the mean relative abundance of bacterial phyla within each season. The sequences that could not be classified into any known phyla were assigned as “Unclassified,” and the sequences with low mean relative abundance (<0.1%) were assigned as “Other phyla.”

**FIGURE 4:**
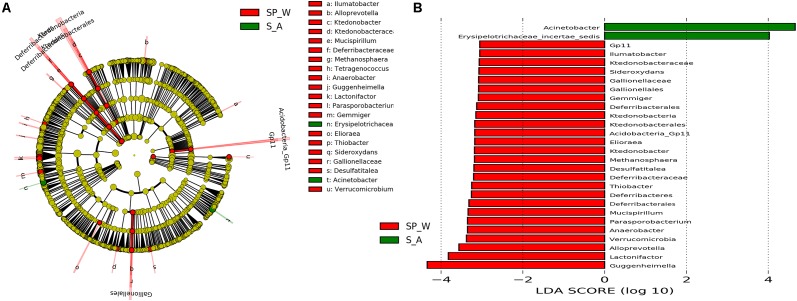
LEfSe analysis of bacterial composition of FMD between S_A (summer and autumn) and SP_W (spring and winter) seasons. The cladogram **(A)** showing the differences in relative abundance of taxa at five levels, and the red and green circles mean differences in relative abundance and yellow circles mean non-significant differences. The LDA histogram **(B)** showing the differences in relative abundance of taxa, and the length of the bar column represents the LDA score.

Gut bacterial communities showed clear seasonal differences in Firmicutes and Bacteroidetes abundances (**Figure 3**). The relative abundance of the bacterial phyla across the four seasons are shown in **Table 1**. Further, ANOVA indicated significant effects of season on the abundances of Firmicutes and Bacteroidetes, whereas no such effects were observed for the abundances of Proteobacteria, Verrucomicrobia, and Actinobacteria (**Table 1**). The abundances of Firmicutes in FMD during spring and winter were significantly higher than during summer and autumn (**Table 2**). In contrast, the Bacteroidetes had markedly lower abundances in FMD during spring and winter compared to summer and autumn (**Table 2**). The Firmicutes–Bacteroidetes ratio at spring (7.74 ± 2.48) and winter (6.44 ± 2.84) was significantly higher than at summer (2.77 ± 0.99) and autumn (2.59 ± 1.02, **Table 2**). FMD in summer and autumn were fed with same plant leaves, as well as in spring and winter. Further, alpha-diversity analysis showed no significant differences between summer and autumn, and spring and winter, so we classified the four seasons into two groups in the following LEfSe analysis. LEfSe analysis indicated statistical differences in 28 taxa between S_A seasons (summer and autumn) and SP_W seasons (spring and winter) (**Figure 4**). The dominant genera among the four seasons comprised *Sporobacter* (spring, 8.68%; summer, 6.68%; autumn, 10.03%; winter, 7.55%), *Bacteroides* (spring, 6.77%; summer, 6.97%; autumn, 6.98%; winter, 7.96%), *Clostridium* XlVa (spring, 5.39%; summer, 6.99%; autumn, 6.07%; winter, 5.81%), *Clostridium* IV (spring, 5.30%; summer, 4.69%; autumn, 6.27%; winter, 4.70%), *Paraprevotella* (spring, 3.18%; summer, 2.31%; autumn, 3.18%; winter, 3.14%), *Alistipes* (spring, 3.16%; summer, 2.87%; autumn, 2.90%; winter, 3.28%), and *Akkermansia* (spring, 2.30%; summer, 4.51%; autumn, 3.21%; winter, 1.02%). Though no significant differences in the relative abundances of these genera were identified among four seasons (**Supplementary Figure S6**), the *Akkermansia* showed significant higher abundance in fresh leave seasons (summer and autumn) than in dry leave seasons (spring and winter) after we pooled the data (*t* = 2.13, *df* = 21.13, *p* = 0.045).

**Table 1:** The relative abundance (% ± SD) and ANOVA tests of the effects of season on bacterial phyla in 32 samples.

Phyla	Spring	Summer	Autumn	Winter	Significance
Firmicutes	77.63 ± 4.94	64.25 ± 5.75	63.76 ± 8.22	75.41 ± 6.28	*F* = 5.69, df = 3, *p* = 0.004
Bacteroidetes	11.51 ± 3.46	25.99 ± 6.78	28.04 ± 5.84	14.83 ± 5.54	*F* = 11.69, df = 3, *p* < 0.001
Proteobacteria	3.50 ± 2.66	3.36 ± 3.41	1.93 ± 1.12	2.59 ± 1.07	H = 1.95, df = 3, *p* = 0.584
Verrucomicrobia	2.46 ± 1.73	2.23 ± 2.13	3.35 ± 2.25	1.58 ± 1.04	H = 1.90, df = 3, *p* = 0.594
Actinobacteria	1.45 ± 1.67	1.13 ± 1.21	0.77 ± 0.63	0.89 ± 0.65	H = 0.435, df = 3, *p* = 0.933
Planctomycetes	1.03 ± 0.87	0.99 ± 0.83	0.54 ± 0.49	0.54 ± 0.43	H = 2.032, df = 3, *p* = 0.566
Spirochaetes	0.48 ± 0.35	0.19 ± 0.17	0.39 ± 0.29	0.47 ± 0.38	H = 3.173, df = 3, *p* = 0.366
Lentisphaerae	0.25 ± 0.11	0.19 ± 0.16	0.26 ± 0.14	0.21 ± 0.15	F = 0.288, df = 3, *p* = 0.834
Candidatus Saccharibacteria	0.29 ± 0.27	0.51 ± 0.60	0.17 ± 0.13	0.16 ± 0.12	H = 0.124, df = 3, *p* = 0.989
Synergistetes	0.16 ± 0.22	0.02 ± 0.01	0.03 ± 0.02	0.06 ± 0.07	H = 4.219, df = 3, *p* = 0.239
Acidobacteria	0.12 ± 0.16	0.11 ± 0.16	0.02 ± 0.02	0.68 ± 1.14	H = 1.885, df = 3, *p* = 0.597
Tenericutes	0.02 ± 0.01	0.05 ± 0.04	0.02 ± 0.03	0.04 ± 0.04	H = 1.842, df = 3, *p* = 0.606
Fusobacteria	0.004 ± 0.006	0.003 ± 0.004	0.003 ± 0.004	0.019 ± 0.030	H = 0.696, df = 3, *p* = 0.874
Euryarchaeota	0.05 ± 0.03	0.07 ± 0.04	0.02 ± 0.02	0.10 ± 0.13	H = 6.219, df = 3, *p* = 0.101
Crenarchaeota	0	0.001 ± 0.002	0	0.001 ± 0.002	H = 2.067, df = 3, *p* = 0.559
Gemmatimonadetes	0.01 ± 0.02	0.02 ± 0.02	0.003 ± 0.004	0.09 ± 0.15	H = 0.084, df = 3, *p* = 0.994
Thermotogae	0.001 ± 0.002	0	0.001 ± 0.002	0.016 ± 0.028	H = 1.075, df = 3, *p* = 0.783
Nitrospirae	0.011 ± 0.017	0.004 ± 0.006	0.001 ± 0.002	0.025 ± 0.041	H = 0.733, df = 3, *p* = 0.866
SR1	0	0	0	0.003 ± 0.004	H = 3.000, df = 3, *p* = 0.392
Fibrobacteres	0	0.001 ± 0.002	0	0.011 ± 0.020	H = 2.069, df = 3, *p* = 0.558
Chloroflexi	0.09 ± 0.12	0.06 ± 0.07	0.01 ± 0.01	0.31 ± 0.51	H = 1.306, df = 3, *p* = 0.728
Thaumarchaeota	0	0	0	0.01 ± 0.02	H = 3.000, df = 3, *p* = 0.392
Latescibacteria	0.004 ± 0.006	0.003 ± 0.004	0	0.005 ± 0.009	H = 2.308, df = 3, *p* = 0.511
Caldiserica	0	0	0	0.001 ± 0.002	H = 3.000, df = 3, *p* = 0.392
Candidate division WPS-2	0.004 ± 0.006	0.005 ± 0.008	0.001 ± 0.002	0.020 ± 0.035	H = 0.778, df = 3, *p* = 0.855
Chlamydiae	0.009 ± 0.013	0.003 ± 0.004	0.001 ± 0.002	0.013 ± 0.022	H = 0.675, df = 3, *p* = 0.879
Deinococcus-Thermus	0.010 ± 0.018	0.011 ± 0.017	0.003 ± 0.004	0.011 ± 0.020	H = 0.604, df = 3, *p* = 0.895
Cloacimonetes	0.003 ± 0.004	0	0.003 ± 0.004	0.003 ± 0.004	H = 2.385, df = 3, *p* = 0.497
Aminicenantes	0	0	0	0.001 ± 0.002	H = 3.000, df = 3, *p* = 0.392
Parcubacteria	0.019 ± 0.026	0.008 ± 0.009	0.001 ± 0.002	0.074 ± 0.127	H = 1.781, df = 3, *p* = 0.619
Hydrogenedentes	0.003 ± 0.004	0	0	0.001 ± 0.002	H = 3.920, df = 3, *p* = 0.270
Elusimicrobia	0.01 ± 0.01	0.01 ± 0.02	0.01 ± 0.01	0.01 ± 0.01	H = 0.904, df = 3, *p* = 0.825
Atribacteria	0	0	0	0.004 ± 0.007	H = 3.000, df = 3, *p* = 0.392
BRC1	0.001 ± 0.002	0	0	0.004 ± 0.007	H = 2.069, df = 3, *p* = 0.558
Candidate division WPS-1	0.004 ± 0.006	0.006 ± 0.009	0	0.018 ± 0.028	H = 2.361, df = 3, *p* = 0.501
Armatimonadetes	0.003 ± 0.004	0.001 ± 0.002	0	0.016 ± 0.028	H = 2.084, df = 3, *p* = 0.555
Ignavibacteriae	0.004 ± 0.006	0.003 ± 0.004	0	0.014 ± 0.024	H = 2.016, df = 3, *p* = 0.569
Chlorobi	0	0	0	0.003 ± 0.004	H = 3.000, df = 3, *p* = 0.392
Deferribacteres	0.013 ± 0.014	0	0	0.003 ± 0.004	H = 3.920, df = 3, *p* = 0.270
Unclassified	0.845 ± 0.225	0.678 ± 0.123	0.645 ± 0.156	1.011 ± 0.445	H = 4.337, df = 3, *p* = 0.227

*The significances of Firmicutes, Bacteroidetes, and Lentisphaerae were determined using the one-way ANOVA, whereas the non-parametric Kruskal–Wallis ANOVA was used to examine the significances of all other phyla.*

**Table 2:** The tests of the effects of season on the relative abundance of Firmicutes, Bacteroidetes, and Firmicutes to Bacteroidetes ratio (F: B ratio).

Items	Seasons	Summer	Autumn	Winter
Firmicutes (*F* = 5.69, *df* = 3)	Spring	*p* = 0.004	*p* = 0.003	*p* = 0.610
	Summer		*p* = 0.911	*p* = 0.015
	Autumn			*p* = 0.012
Bacteroidetes (*F* = 11.69, *df* = 3)	Spring	*p* < 0.001	*p* < 0.001	*p* = 0.332
	Summer		*p* = 0.549	*p* = 0.003
	Autumn			*p* = 0.001
F: B ratio (*df* = 3)	Spring	H = 14.38, *p* = 0.013	H = 16.50, *p* = 0.003	H = 3.63, *p* = 0.440
	Summer		H = 2.13, *p* = 0.651	H = 10.75, *p* = 0.022
	Autumn			H = 12.88, *p* = 0.006

*The significances of Firmicutes and Bacteroidetes were determined using the one-way ANOVA, and the LSD test was used to apply *post hoc* tests. The significances of F: B ratio were determined using the non-parametric Kruskal–Wallis ANOVA.*

**FIGURE 5:**
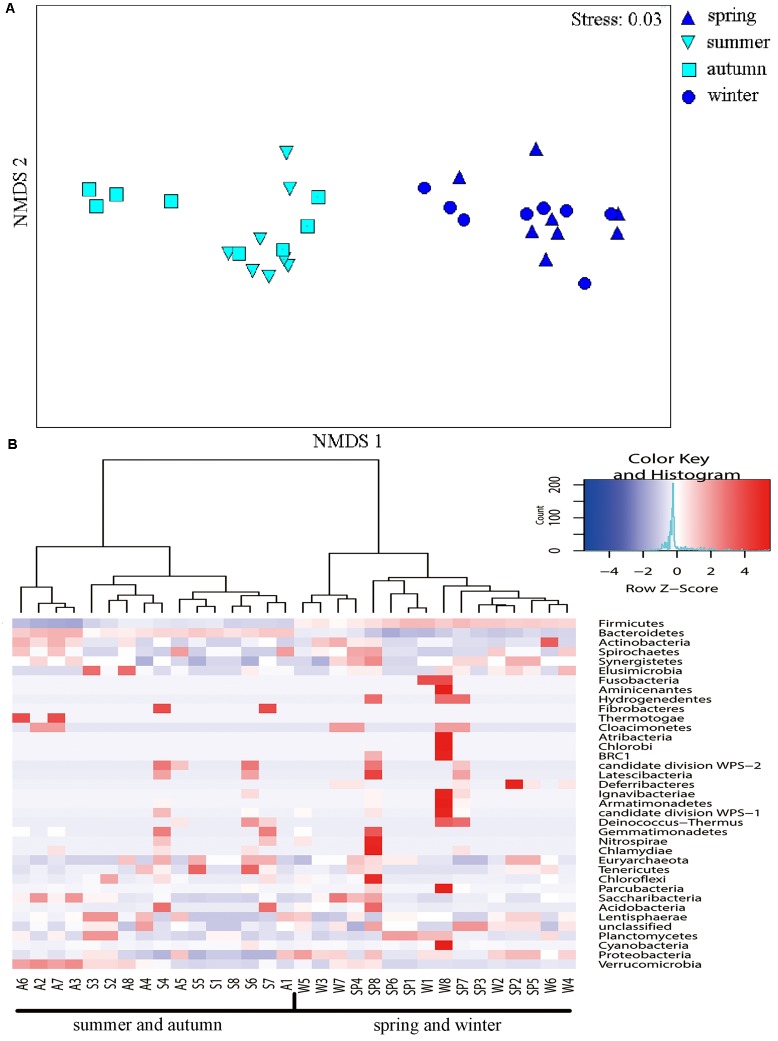
Non-metric multidimensional scaling (NMDS) and Heatmap analysis of distance between different seasons. **(A)** The NMDS plots showing the distance between the samples, based on dissimilarity in OTU composition of each sample was calculated using the Bray–Curtis dissimilarity index. Each point represents a different sample and a greater distance between two points infers a higher dissimilarity between them. **(B)** Heatmap analysis of the bacterial distribution among the 32 samples based on hierarchical clustering (Bray–Curtis distance metric and complete clustering method). Each row represents a bacterial phylum where columns represent the 32 individual samples. The values in the Heatmap represent the square root-transformed relative percentage of each bacterial phylum.

The NMDS ordination indicated obvious differences among communities from individuals in spring–winter and summer–autumn seasons (**Figure 5A**). Accordingly, ANOSIM indicated significant differences in bacterial community composition between spring and summer (*R* = 0.89, *p* = 0.001), spring and autumn (*R* = 0.98, *p* = 0.001), winter and summer (*R* = 0.97, *p* = 0.001), and winter and autumn (*R* = 0.91, *p* = 0.001). Further, ANOSIM also indicated significant differences in microbial communities between summer and winter (*R* = 0.23, *p* = 0.034), although the NMDS ordination showed some overlap among individuals between these two seasons (**Figure 5A**). Hierarchical cluster analysis of phylum-level differences showed that microbial communities in spring and winter samples belonged to one group, whereas communities from summer and autumn samples belonged to another group. Thus, the cluster analysis did not clearly distinguish samples between summer and autumn, in addition to spring and winter (**Figure 5B**).

## Discussion

The present study investigated whether FMD gut microbial communities shifted with the seasonal changes in the host diet, in order to assess how these shifts help FMD meet nutritional demands. The results demonstrated that (1) the effects of seasonal dietary shifts in the core FMD gut bacterial populations are limited; (2) there are significant differences in the composition and Alpha-diversity of FMD gut microbiota across seasons, especially regarding the Firmicutes–Bacteroidetes ratio, and these results were supported by Beta-diversity comparisons of microbial communities.

Diet is the one of the critical factors in shaping gut microbial communities (Scott et al., 2013), and especially in the digestive tract of ruminants (particularly in the rumen), which is a suitable environment to ferment high fiber plants to produce nutrients for animal. However, ruminants cannot produce the essential cellulolytic enzymes, which must be produced by colonized gut microbes (Krause et al., 2003). Thus, the ratio of fiber and starch in diets can remarkably affect the physiological state of digestive tract in ruminants, and eventually lead to differentiated fecal-associated microflora (Belanche et al., 2012). Our results indicated that the core bacterial phyla of FMD belonged to the phyla Firmicutes and Bacteroidetes, which is consistent with previous studies of musk deer (Hu et al., 2017; Li et al., 2017) and other ruminants (Henderson et al., 2015; Delgado et al., 2017; Guan et al., 2017; O’Donnell et al., 2017). Firmicutes and Bacteroidetes are ubiquitously distributed phyla in the gut microbial communities of many mammals, indicating their ecological and functional significance in the digestive tract (Ley et al., 2008; Shanks et al., 2011). An important function of Firmicutes is the ability to degrade fibers into short chain fatty acids (SCFAs) that can be used by ruminants, while Bacteroidetes are primarily responsible for the degradation of carbohydrates, fats and proteins (Jami et al., 2013). The core bacteria of FMD at the different taxonomic levels were relatively stable across the four seasons, suggesting that the effects of season on gut microbiota were limited. Previous studies (Henderson et al., 2015) on ruminants from different countries found a relatively stable core bacterial microbiome across 32 animal species regardless of dietary variations. These results both indicated that a stable core gut microbiome was present in ruminants, and environmental factors might affect the abundance of these core bacterial groups (Weimer, 2015).

Though the core bacteria of FMD is stable across seasons, the composition of bacterial communities is sensitive to diverse perturbations, including dietary changes corresponding to seasonal changes (David et al., 2014). We observed a substantial effect of presumptive seasonal nutrient variation on gut bacterial composition at the phylum level. The Beta-diversity comparisons revealed obvious differences between samples from seasons where fresh leaves were used as food (summer and autumn) and seasons with dry leaf food (spring and winter). Further, LEfSe analysis identified many taxa with significantly differential representation among the groups. However, the differences in relative abundances of the five most abundant genera were not significant among the four seasons. Many OTUs could not be classified into a known genus, and thus, differences in the relative abundance of genera could be misleading. Consequently, assessing seasonal variation by phylum-level abundance differences could better indicate bacterial community differences across groups, as phylum-specific differences in functionalities (e.g., digestion of fiber, starch, and proteins) are evident (Springer et al., 2017). In this study, FMD were fed fresh leaves with more starch, protein, and lactate content in the summer and autumn, whereas the winter and early spring diets mainly included dry leaves with more fiber (Wang W.X. et al., 2013; Wang Y.Q. et al., 2013). The dietary change resulted in different gut microbial composition at phylum level, which was in line with previous study (Fernando et al., 2010). The observed variation in Firmicutes of FMD was mainly attributed to *Ruminococcaceae* and *Lachnospiraceae*. The *Ruminococcaceae* and *Lachnospiraceae* are efficient fermenters of fibrous compounds such as cellulose or xylan, and produce SCFAs (Flint et al., 2012). SCFAs can reduce intestinal pH, making gut conditions more unfavorable for Bacteroidetes (Duncan et al., 2009). Nevertheless, the results did not indicate a significantly shift in Proteobacteria, this may be because the animals were managed over the winter to prevent a loss of body condition. At the genus level, the higher abundance of *Akkermansia* might indicate a more healthy gut microecosystem, as it plays a probiotic role by degrading intestinal mucin, regulating the thickness of intestinal mucus and maintaining intestinal barrier integrity (Zhou, 2017).

The Firmicutes–Bacteroidetes ratio is an important attribute of microbial communities. A higher Firmicutes–Bacteroidetes ratio from samples collected in seasons where dry leaves were used as food was observed in this dataset. Further, several studies of both model (mouse, Turnbaugh et al., 2008) and wild (monkey, Amato et al., 2015) animals suggest a functional association of this microbiota characteristic to increased energy extraction from high fiber diets. Variation in the relative abundances of Bacteroidetes and Firmicutes likely reflects the adaptation of FMD to the quality and consistency of seasonal diets, wherein microbial fermentation might compensate for reduced energy intake during seasons with limited food resources (Li et al., 2013; Amato et al., 2015). The gut microbial communities during the seasons with dry leaf feeding (spring and winter) was more diverse than in the seasons with fresh leaf feeding (summer and autumn), as indicated by statistically significant differences of alpha diversity indices (Shannon and Simpson). In general, high-fiber diets can increase gut microbial diversity as revealed by several previous studies (De Filippo et al., 2010; Pitta et al., 2010). Moreover, the use of anthelmintic drugs can also significantly affect intestinal microbial diversity (Greenwood et al., 2014; Minter et al., 2016), though microbial communities can recover more or less within a few days or weeks after drug withdrawal (Dethlefsen et al., 2008). At the FMD breeding center, animals are dewormed several times per year, mostly in the summer and early autumn. Here, we suppose that the use of antibiotics can reduce the gut microbial diversity in FMD, along with seasonal dietary changes.

Using high-throughput sequencing technique based on gut microbial 16S rRNA genes, our results provide evidence that the composition of FMD gut microbiota are shaped by variation in seasonal dietary shifts. These dietary shifts are associated with changes in food type (fresh vs. dry leaves) in captive FMD. Consequently, this seasonal variation in gut microbiota may facilitate fiber utilization by reinforcing microbial robustness in seasons with nutrient-deficient diets. The results of this study highlight the important role of gut microbiota in the nutritional ecology of captive FMD and provide vital baseline data for understanding the seasonally dynamic relationships between FMD and gut microbiota. Although, these seasonal shifts suggest an adaptation or adjustment of the gut microbiota to the host diet, future studies are necessary to further investigate the mechanisms underlying these interactions.

## Author Contributions

XH and GL carried out the sample collection, DNA extraction, data analysis, and drafted the manuscript. YL participated in drafting the manuscript and analysis. YW and SL participated in the sample collection and DNA extraction. SQL participated in the sample collection and data analysis. YZ and DH who are the corresponding authors, conceived of the study and participated in its design and coordination and helped to draft the manuscript. All authors read and approved the final manuscript.

## Conflict of Interest Statement

The authors declare that the research was conducted in the absence of any commercial or financial relationships that could be construed as a potential conflict of interest.

## Footnotes

1 http://www.r-project.org/

2 http://huttenhower.sph.harvard.edu/galaxy/

## Supplementary Material

FIGURE S1 The rarefaction curves of OTUs **(A)** and Shannon index **(B)** for the 32 samples. SP1–SP8 represent the samples collected from forest musk deer at spring, S1–S8 represent the samples collected at summer, A1–A8 represent the samples collected at autumn, W1–W8 represent the samples collected at winter.

FIGURE S2 The pie diagrams of core bacteria at genus **(A)**, family **(B)**, order **(C)**, and class **(D)** levels for forest musk deer in spring.

FIGURE S3 The pie diagrams of core bacteria at genus **(A)**, family **(B)**, order **(C)**, and class **(D)** levels for forest musk deer in summer.

FIGURE S4 The pie diagrams of core bacteria at genus **(A)**, family **(B)**, order **(C),** and class **(D)** levels for forest musk deer in autumn.

FIGURE S5 The pie diagrams of core bacteria at genus **(A)**, family **(B)**, order **(C)**, and class **(D)** levels for forest musk deer in winter.

FIGURE S6 The differences in relative abundance of the top five genera (except the unclassified bacteria) among four seasons.

TABLE S1 List of the barcodes used to tag each PCR product analyzed in the study, and the cleaned sequence number and length for each sample. SP1–SP9 represent the samples collected at spring, S1–S9 represent the samples collected at summer, A1–A9 represent the samples collected at autumn, W1–W9 represent the samples collected at winter.

TABLE S2 Number of OTUs, estimated OTU richness (ACE and Chao1), diversity index (Shannon and Simpson) and estimated sample Coverage for the different samples. SP1–SP9 represent the samples collected at spring, S1–S9 represent the samples collected at summer, A1–A9 represent the samples collected at autumn, W1–W9 represent the samples collected at winter.
