# Supplementary figures and images for: High-Throughput Analysis Reveals Seasonal Variation of the Gut Microbiota Composition Within Forest Musk Deer (Moschus berezovskii)
Source: Front Microbiol. 2018 Jul 26;9:1674. doi: 10.3389/fmicb.2018.01674 (PMC6070636; doi:10.3389/fmicb.2018.01674)

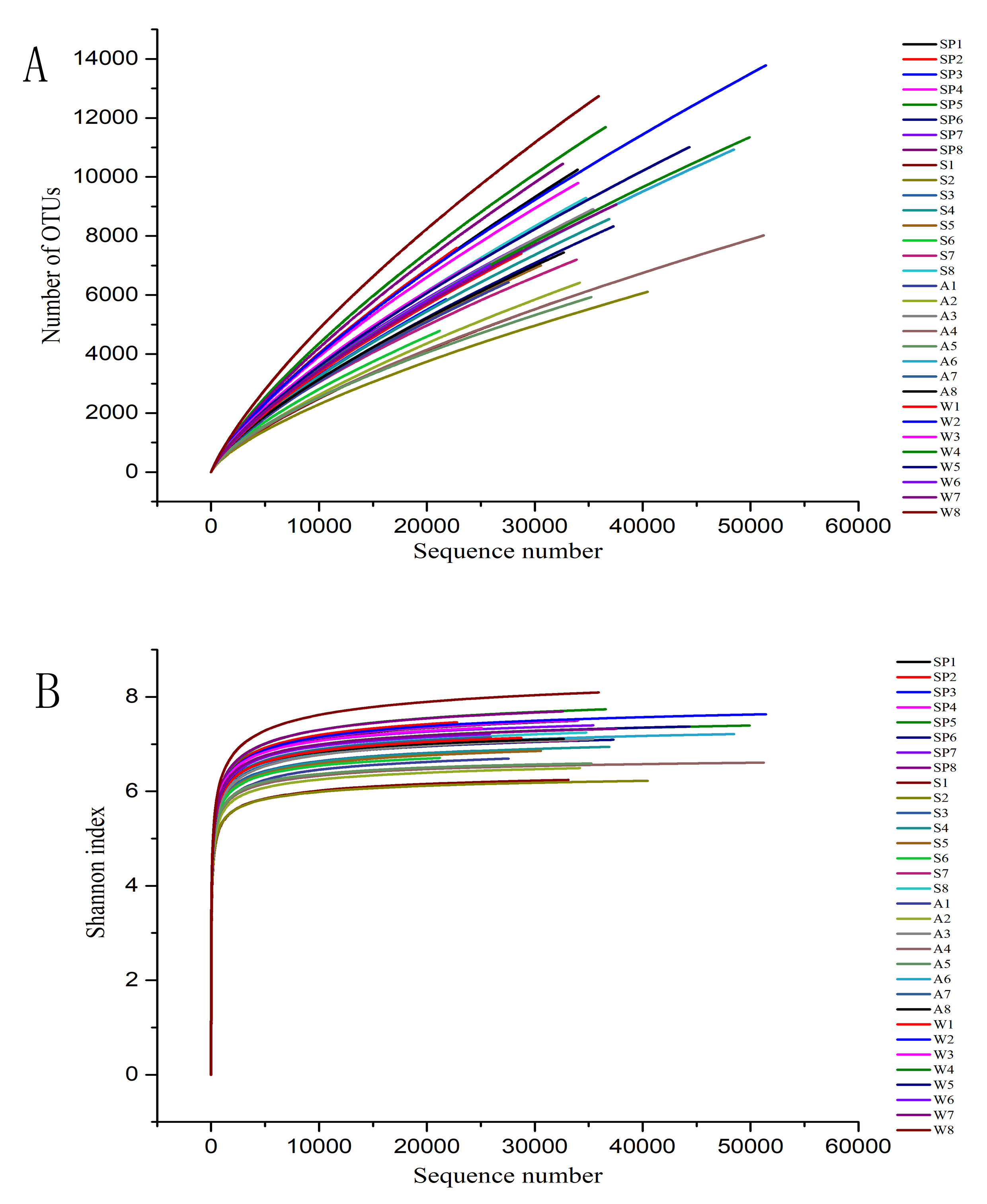

Supplement: FIGURE S1 — The rarefaction curves of OTUs (A) and Shannon index (B) for the 32 samples. SP1–SP8 represent the samples collected from forest musk deer at spring, S1–S8 represent the samples collected at summer, A1–A8 represent the samples collected at autumn, W1–W8 represent the samples collected at winter. [file Image_1.TIF]
