# Supplementary material for: High-Throughput Analysis Reveals Seasonal Variation of the Gut Microbiota Composition Within Forest Musk Deer (Moschus berezovskii)
Source: Front Microbiol. 2018 Jul 26;9:1674. doi: 10.3389/fmicb.2018.01674 (PMC6070636; doi:10.3389/fmicb.2018.01674)
Supplement: TABLE S2 — Number of OTUs, estimated OTU richness (ACE and Chao1), diversity index (Shannon and Simpson) and estimated sample Coverage for the different samples. SP1–SP9 represent the samples collected at spring, S1–S9 represent the samples collected at summer, A1–A9 represent the samples collected at autumn, W1–W9 represent the samples collected at winter. [file Table_2.DOCX]

**Table S2**

Number of OTUs, estimated OTU richness (ACE and Chao1), diversity index (Shannon and Simpson) and estimated sample Coverage for the different samples. SP1-SP9 represent the samples collected at spring, S1-S9 represent the samples collected at summer, A1-A9 represent the samples collected at autumn, W1-W9 represent the samples collected at winter.

| Samples | OTUs | ACE | Chao1 | Shannon | Simpson | Coverage (%) |
| --- | --- | --- | --- | --- | --- | --- |
| SP1 | 10244 | 85498 | 39562 | 7.49 | 0.0044 | 77.03 |
| SP2 | 7593 | 67128 | 30814 | 7.46 | 0.0042 | 74.39 |
| SP3 | 5853 | 45803 | 22093 | 7.04 | 0.0041 | 79.94 |
| SP4 | 9799 | 69823 | 33632 | 7.50 | 0.0040 | 78.83 |
| SP5 | 11688 | 78757 | 38470 | 7.74 | 0.0040 | 76.40 |
| SP6 | 8327 | 56748 | 28188 | 7.09 | 0.0038 | 83.61 |
| SP7 | 8900 | 63500 | 29694 | 7.40 | 0.0036 | 81.84 |
| SP8 | 10445 | 83246 | 39323 | 7.69 | 0.0028 | 75.69 |
| S1 | 5910 | 37545 | 19204 | 6.24 | 0.0165 | 87.31 |
| S2 | 6109 | 34300 | 18724 | 6.22 | 0.0116 | 89.60 |
| S3 | 5926 | 40764 | 19586 | 6.86 | 0.0084 | 82.58 |
| S4 | 8574 | 59840 | 29243 | 6.94 | 0.0078 | 82.96 |
| S5 | 7004 | 48127 | 23147 | 6.86 | 0.0069 | 83.41 |
| S6 | 4786 | 32193 | 16938 | 6.71 | 0.0065 | 83.87 |
| S7 | 7197 | 45879 | 23075 | 7.08 | 0.0055 | 85.17 |
| S8 | 9293 | 67289 | 31802 | 7.24 | 0.0052 | 80.21 |
| A1 | 6431 | 45874 | 22259 | 6.69 | 0.0123 | 83.07 |
| A2 | 6417 | 39952 | 20094 | 6.49 | 0.0089 | 86.59 |
| A3 | 8907 | 66468 | 31587 | 7.11 | 0.0079 | 81.46 |
| A4 | 8021 | 48883 | 24963 | 6.61 | 0.0072 | 89.05 |
| A5 | 5928 | 33555 | 18548 | 6.59 | 0.0066 | 88.63 |
| A6 | 10928 | 73096 | 35793 | 7.21 | 0.0059 | 83.57 |
| A7 | 7050 | 54995 | 25439 | 7.15 | 0.0053 | 79.80 |
| A8 | 7442 | 52228 | 25052 | 7.11 | 0.0045 | 83.58 |
| W1 | 7405 | 52139 | 26327 | 7.13 | 0.0043 | 81.04 |
| W2 | 13780 | 100315 | 47916 | 7.63 | 0.0041 | 80.21 |
| W3 | 7237 | 55078 | 26755 | 7.34 | 0.0041 | 78.68 |
| W4 | 11340 | 81774 | 39578 | 7.39 | 0.0040 | 83.43 |
| W5 | 11010 | 77351 | 37642 | 7.37 | 0.0040 | 81.70 |
| W6 | 6994 | 53444 | 24246 | 7.21 | 0.0037 | 79.98 |
| W7 | 9068 | 65705 | 30226 | 7.32 | 0.0035 | 82.48 |
| W8 | 12737 | 83698 | 42760 | 8.09 | 0.0024 | 73.95 |
